# Promoting ribosomal incorporation of backbone-modifying nonproteinogenic amino acids into nascent peptides by ATP-binding cassette family-F proteins and EF-P

**DOI:** 10.1093/nar/gkaf446

**Published:** 2025-05-22

**Authors:** Takayuki Katoh, Hiroaki Suga

**Affiliations:** Department of Chemistry, Graduate School of Science, The University of Tokyo, 7-3-1 Hongo, Bunkyo-ku, Tokyo 113-0033, Japan; Department of Chemistry, Graduate School of Science, The University of Tokyo, 7-3-1 Hongo, Bunkyo-ku, Tokyo 113-0033, Japan

## Abstract

In the past two decades, tremendous efforts for increasing the efficiency of ribosomal incorporation of backbone-modifying nonproteinogenic amino acids (npAAs) have been made and given significant successes. For instance, the use of an engineered body sequence of transfer RNA (tRNA), known as tRNA^Pro1E2^, that efficiently recruits EF-Tu and EF-P significantly improves consecutive incorporation of npAAs, giving a notion that certain protein factors paired with right tRNAs can enhance their incorporation efficiency. However, the consecutive incorporation of certain npAAs, e.g.*N*-methyl-l-leucine, remains more challenging. Here we have explored *Escherichia coli* ATP-binding cassette family-F proteins (EttA, Uup, YbiT, and YhsS) and RbbA for a possibility of enhancing the translation efficiency for such npAAs since these proteins are known to alleviate nascent peptide-dependent translation arrest. Indeed, among them the presence of Uup increases the translation level of model peptides bearing two consecutive npAAs by an average of 1.7-fold for 12 kinds of npAAs and that of a macrocyclic peptide bearing d-α-amino, *N*-methyl-l-α-amino, and β-amino acids by 1.8-fold. Moreover, the combination of EF-P and Uup further enhances the incorporation of npAAs charged on tRNA^Pro1E2^, demonstrating a four-fold enhancement for two consecutive incorporations of *N*-methyl-l-leucine.

## Graphical Abstract

**Graphical Abstract:**
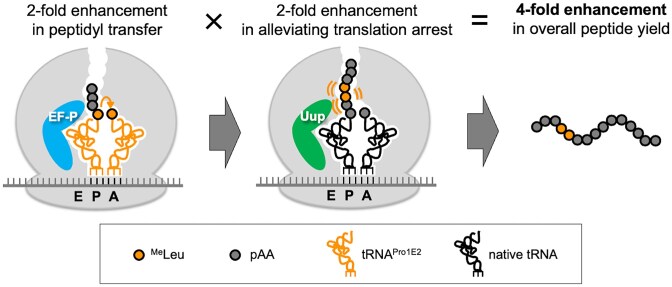

## Introduction

Genetic code reprogramming enables ribosomal incorporation of nonproteinogenic amino acids (npAAs) into peptides [1]. Not only l-α-amino acids with side chain modifications but also backbone-modifying ones, such as d-α-amino acids, α,α-disubstituted amino acids, *N*-methyl-l-α-amino acids, β-amino acids, and γ-amino acids, can be introduced by ribosomal translation [2–7]. Backbone-modifying npAAs often induce unique folding propensities, such as turns and helices, of the resulting peptides by introducing hydrogen bonds, steric hindrance, and conformational constraints, leading to more rigid and defined structures compared with peptides composed solely of proteinogenic amino acids (pAAs) [8–19]. Such peptides tend to show strong binding affinity to target molecules, as well as enhanced proteolytic stability and membrane permeability, making them attractive scaffolds suitable for developing peptide drugs [12, 20–30]. Messenger RNA (mRNA) display-based screening methods, such as the random nonstandard peptides integrated discovery (RaPID) system, can be applied to ribosomally synthesized peptide libraries to screen bioactive peptides containing npAAs [23, 31–33]. However, ribosomal incorporation of backbone-modifying npAAs is much less efficient compared with that of the canonical l-α-amino acids [2, 3, 6, 34, 35]. Multiple and/or consecutive incorporation of the npAAs is even more challenging, often resulting in termination caused by peptidyl-tRNA drop-off from the ribosomal P-site. This has been a major issue in prohibiting the preparation of random peptide libraries containing multiple npAAs in a high quality enough for the screening.

The inefficiency can be primarily attributed to the following reasons: (i) the slow accommodation of aminoacyl-tRNA (npAA-tRNA) onto the ribosomal A-site due to their low binding affinity to EF-Tu and (ii) the slow peptidyl transfer reaction between the P-site peptidyl-tRNA and A-site aminoacyl-tRNA [4, 34–38]. To overcome these challenges, we developed an engineered tRNA, referred to as tRNA^Pro1E2^, that has specific T-stem and D-arm motifs for efficient accommodation and peptidyl transfer by binding to EF-Tu and EF-P, respectively [36]. EF-Tu binds to aminoacyl-tRNA by recognizing the amino acid moiety and the T-stem of aminoacyl-tRNA, but the affinity to the amino acid moiety is low for npAA-tRNA, resulting in slow accommodation [38]. Using tRNA^Pro1E2^, the low affinity can be compensated for by enhancing the affinity of the T-stem moiety, thereby accelerating accommodation. In nature, EF-P enhances Pro incorporation. We demonstrated that in reprogrammed translation systems, incorporation of backbone-modifying npAAs is also enhanced by EF-P [36, 39, 40]. In addition, we reported that the specific D-arm motif derived from tRNA^Pro^ isoacceptors is essential for recognition by EF-P [41]. EF-P binds to a site between E-site and P-site by recognizing the D-arm of P-site peptidyl-tRNA [42]. By introducing the D-arm motif into tRNA^Pro1E2^, incorporation of various npAAs charged on tRNA^Pro1E2^ can be enhanced in the presence of EF-P. Taking advantage of tRNA^Pro1E2^, we developed random peptide libraries containing npAAs, which were then applied to RaPID screening of bioactive peptides [22, 24–30]. However, the consecutive incorporation of certain npAAs, e.g.*N*-methyl-l-leucine, remains to be challenging.

It has been reported that certain nascent peptide sequences, such as poly-Asp/Glu, poly-Arg/Lys, and poly-Pro, interact with the ribosomal exit tunnel [43–45], distorting their peptidyl-tRNA in P-site and locating it at a suboptimal position for the peptidyl transfer reaction, and thus making it difficult to react with the α-amino group of aminoacyl-tRNA in A-site [45]. In addition, the nascent peptides trigger structural rearrangement of the ribosome to cause ribosomal stalling and destabilization [46]. For instance, in poly-Pro incorporation, the *trans* conformation is favored for Pro; however, the resulting nascent peptide bearing poly-Pro with *trans* conformations is unfavorable for going through the ribosomal tunnel [45]. Due to the rigid nature of Pro, the nascent peptide becomes tightly bound to the tunnel, hindering further elongation. Because the backbone-modifying npAAs induce rigid and unique peptide folding, it is possible that nascent peptides bearing npAAs trigger translation arrest and ribosome destabilization by forming unfavorable conformations for passing the ribosomal tunnel.

The *Escherichia coli* ATP-binding cassette family-F (ABC-F) proteins, EttA, Uup, YbiT, and YhsS, are ATPase proteins that bind to ribosomal E-site. ABC-F proteins are known to rescue ribosomes that are stalled by antibiotics [47–52]. By binding to the E-site, ABC-F inserts the interdomain linker between two ATP-binding domains into the peptidyl transferase (PTase) center of the ribosome in an ATP- or NTP-dependent manner and induces rearrangement of the PTase center and the P-site peptidyl-tRNA. Due to this structural change, the stalled ribosome can be rescued. Recently, ABC-F proteins were reported to be involved in alleviating the translation arrest and ribosome destabilization caused by some nascent peptide sequences [53–55]. Of these ABC-F proteins, Uup resolves poly-Pro-dependent arrest [53]. EttA effectively alleviates ribosome destabilization caused by polyacidic sequences [53, 54]. Structural analysis of EttA revealed that, by binding to ribosomal E-site, it interacts with and stabilizes P-site peptidyl-tRNA [56, 57]. Similarly, YbiT is effective for polybasic and polyacidic sequences, and YhsS for the SecM translation arrest sequence [51, 53]. Although how ABC-F proteins resolve translation arrest is not fully clear, we have given a thought to utilize them for alleviating the translation arrest caused by the incorporation of the backbone-modifying npAAs. In addition to the ABC-F proteins, we have also considered RbbA to use since it is an E-site binder ATPase protein, and it has been previously suggested that RbbA promotes the release of E-site deacyl-tRNA and accommodation of aminoacyl-tRNA onto A-site. It has been shown that RbbA stimulates poly-Phe synthesis [58]. Here, our work has focused on studies of ABC-F proteins and/or RbbA in the context of *in vitro* translation of npAA-containing model peptides and explored consecutive and/or multiple incorporations of d-α-amino acids, α,α-disubstituted amino acids, *N*-methyl-l-α-amino acids, and β-amino acids.

## Materials and methods

### Preparation of tRNA and flexizyme

DNA templates for the transcription of tRNAs and flexizymes (dFx and eFx) were prepared by extension reaction followed by polymerase chain reaction (PCR) using the forward and reverse primer pairs shown in Supplementary Table S1. The PCR products were purified by phenol/chloroform extraction and ethanol precipitation. The resulting DNAs have a T7 promoter preceding the tRNA or flexizyme sequence. Transcription of flexizyme was conducted at 37°C overnight in a 5 ml reaction mixture containing 40 mM Tris–HCl (pH 8.0), 5 mM NTP mix, 22.5 mM MgCl_2_, 1 mM dithiothreitol (DTT), 1 mM spermidine, 0.01% Triton X-100, 0.04 U/μl RNasin RNase Inhibitor (Promega), and 0.12 μM T7 RNA polymerase. For tRNA transcription, 5 mM guanosine monophosphate was added to the reaction mixture, and the NTP mix concentration was reduced to 3.75 mM. The resulting RNAs were treated with RQ1 DNase (Promega) for 30 min at 37°C to remove DNAs and purified using an 8% (tRNAs) or 12% (flexizymes) polyacrylamide gel containing 6 M urea.

### Aminoacylation of tRNA

The npAAs were preactivated as 3,5-dinitrobenzyl ester (DBE) or cyanomethyl ester (CME), as described [59, 60]. β^3^-Homophenylglycine (βPhg), β^3^-homomethionine (βMet), β^3^-homoglutamine (βGln), (1*R*,2*R*)-2-aminocyclopentane carboxylic acid (ACPC), (1*R*,2*S*)-2-ACPC, (1*S*,2*S*)-2-ACPC, d-alanine (d-Ala), d-cysteine (d-Cys), 2-aminoisobutyric acid (Aib), *N*-methyl-l-leucine (^Me^Leu), *N*-methyl-l-aspartic acid (^Me^Asp), and *N*-methyl-l-valine (^Me^Val) were activated as DBE forms. *N*-Chloroacetyl-d-tyrosine (^ClAc^ d-Tyr) was activated as a CME form. Aminoacylation of tRNA was performed at 0°C in reaction mixtures containing 50 mM HEPES–KOH (pH 7.5) or Bicine–KOH (pH 9.0), 200 mM MgCl_2_, 20% DMSO, 25 μM dFx or eFx, 25 μM tRNA, and 5 mM activated amino acids. dFx was used for DBEs and eFx for CMEs. MgCl_2_ concentration was increased to 600 mM when using eFx/CME. Reaction pH was 9.0 for β-amino acids and 7.5 for the others. Reaction time was 2 h ford-Ala, Aib, and ^ClAc^ d-Tyr; 6 h ford-Cys and ^Me^Leu; and 24 h for βPhg, βMet, βGln, 2-ACPC, ^Me^Asp, and ^Me^Val. The resulting aminoacyl-tRNAs were recovered by ethanol precipitation, washed with 70% ethanol, and dissolved in 1 mM sodium acetate (pH 5.2).

### Preparation of EttA, Uup, YbiT, YhsS, and RbbA

The genes encoding *E. coli* EttA and RbbA were cloned into a modified pET28a vector that has a PreScission Protease recognition site in place of the original thrombin recognition site. We cloned a truncated form of RbbA that lacks the C-terminal transmembrane domain, as described [58], because full-length RbbA is unstable and difficult to overexpress and purify. The truncated form is reportedly stable and active. The genes encoding Uup, YbiT, and YhsS were cloned into pET28a-TEV vector. To express Uup, YbiT, and YhsS, modified pET28a or pET28a-TEV bearing each gene was introduced into *E. coli* BL21 (DE3) cells. The cells were cultured in LB medium with 0.5 mM isopropyl β-d-1-thiogalactopyranoside for 2–3 h at 37°C and lysed by sonication. The lysate was applied to a His-TALON crude (5 ml) column (Cytiva). The column was washed with buffer A [20 mM Tris–HCl (pH 8.0), 200 mM NaCl, 10 mM imidazole, and 1 mM DTT]. Then, His-tagged proteins were eluted with buffer A containing 300 mM imidazole. Turbo3C protease or TurboTEV protease was added to the eluate to cleave the His-tag and dialyzed against buffer A at 4°C overnight. The sample was applied to a His-TALON crude column, and the flow-through fraction was recovered and concentrated using Amicon Ultra Centrifugal Filters (Merck Millipore). The resulting proteins were analyzed by 10% sodium dodecyl sulfate–polyacrylamide gel electrophoresis (SDS–PAGE) (Supplementary Fig. S1).

### Translation of model peptides

Translation was performed at 37°C for 30 min using an *E. coli* reconstituted *in vitro* translation system, referred to as the Flexible *In vitro*Translation (FIT) system [23], consisting of the following reagents: 50 mM HEPES–KOH (pH 7.6), 100 mM potassium acetate, 12.3 mM magnesium acetate, 2 mM ATP, 2 mM GTP, 1 mM CTP, 1 mM UTP, 20 mM creatine phosphate, 0.1 mM 10-formyl-5,6,7,8-tetrahydrofolic acid, 2 mM spermidine, 1 mM DTT, 1.5 mg/ml *E. coli* total tRNA, 1.2 μM *E. coli* ribosome, 0.6 μM methionyl-tRNA formyltransferase, 2.7 μM IF1, 3 μM IF2, 1.5 μM IF3, 0.1 μM EF-G, 20 μM EF-Tu/Ts, 5 μM EF-P, 0.25 μM RF2, 0.17 μM RF3, 0.5 μM RRF, 4 μg/ml creatine kinase, 3 μg/ml myokinase, 0.1 μM inorganic pyrophosphatase, 0.1 μM nucleotide diphosphate kinase, 0.1 μM T7 RNA polymerase, 0.13 μM AspRS, 0.09 μM GlyRS, 0.11 μM LysRS, 0.03 μM MetRS, 0.02 μM TyrRS, 0.5 mM each of Asp, Lys, Met, and Tyr, 25 μM each pre-charged aminoacyl-tRNA, and 0.5 μM DNA template. EttA, Uup, YbiT, YhsS, and RbbA were added as required. For the translation of rP1–^Me^Asp, EF-Sep was included. For the translation of rP3, 10-formyl-5,6,7,8-tetrahydrofolic acid was excluded.

For matrix-assisted laser desorption/ionization-time of flight mass spectrometry (MALDI-TOF MS)-based analysis of the peptides, 2.5 μl of the translation reaction mix was diluted with an equal volume of 2× TBS buffer [100 mM Tris–HCl (pH 7.6) and 300 mM NaCl] and incubated with anti-FLAG antibody agarose gel (Sigma) for 15 min at room temperature. The beads were washed with 1× TBS buffer [50 mM Tris–HCl (pH 7.6) and 150 mM NaCl] twice and eluted with 0.1% trifluoroacetic acid. The eluent was applied to SPE C-tip (Nikkyo Technos) for desalting and eluted with 1.0 μl of 80% acetonitrile/0.5% acetic acid solution containing 50% saturated (*R*)-cyano-4-hydroxycinnamic acid. MS analysis was performed using UltrafleXtreme (Bruker Daltonics) in reflector/positive mode. Peptide calibration standard II (Bruker Daltonics) was used for external mass calibration.

For quantification of translated peptides, 0.05 mM [^14^C]-Asp was added to the translation solution in place of cold Asp. An equal volume of stop solution [0.9 M Tris–HCl (pH 8.45), 8% SDS, 30% glycerol, and 0.001% xylene cyanol] was added to the translation solution and incubated at 95°C for 2 min. Then, the peptides were analyzed by 15% tricine SDS–PAGE and autoradiography using Typhoon FLA 7000 (Cytiva). The translation level of the peptides was quantified by the radioisotope intensity of the peptide relative to total [^14^C]-Asp intensity included in the reaction mixture.

## Results

### Ribosomal translation of model peptides containing consecutive npAAs in the presence of ABC-F proteins and RbbA

**Figure 1.**
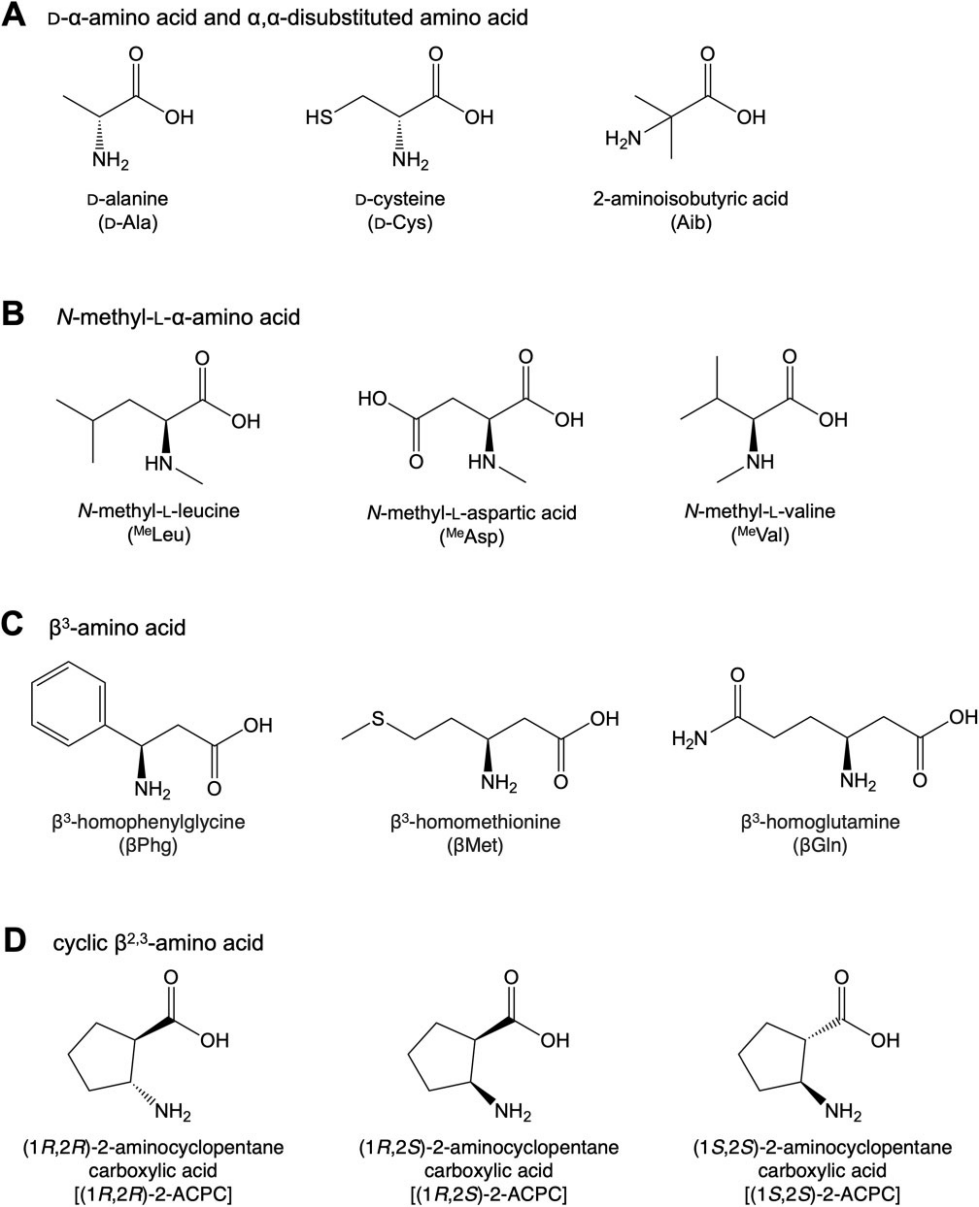
Chemical structures of the nonproteinogenic amino acids tested in this study. (**A**) d-α-Amino acid and α,α-disubstituted amino acid, (**B**) *N*-methyl-l-α-amino acid, (**C**) β^3^-amino acid, and (**D**) cyclic β^2,3^-amino acid.

**Figure 2.**
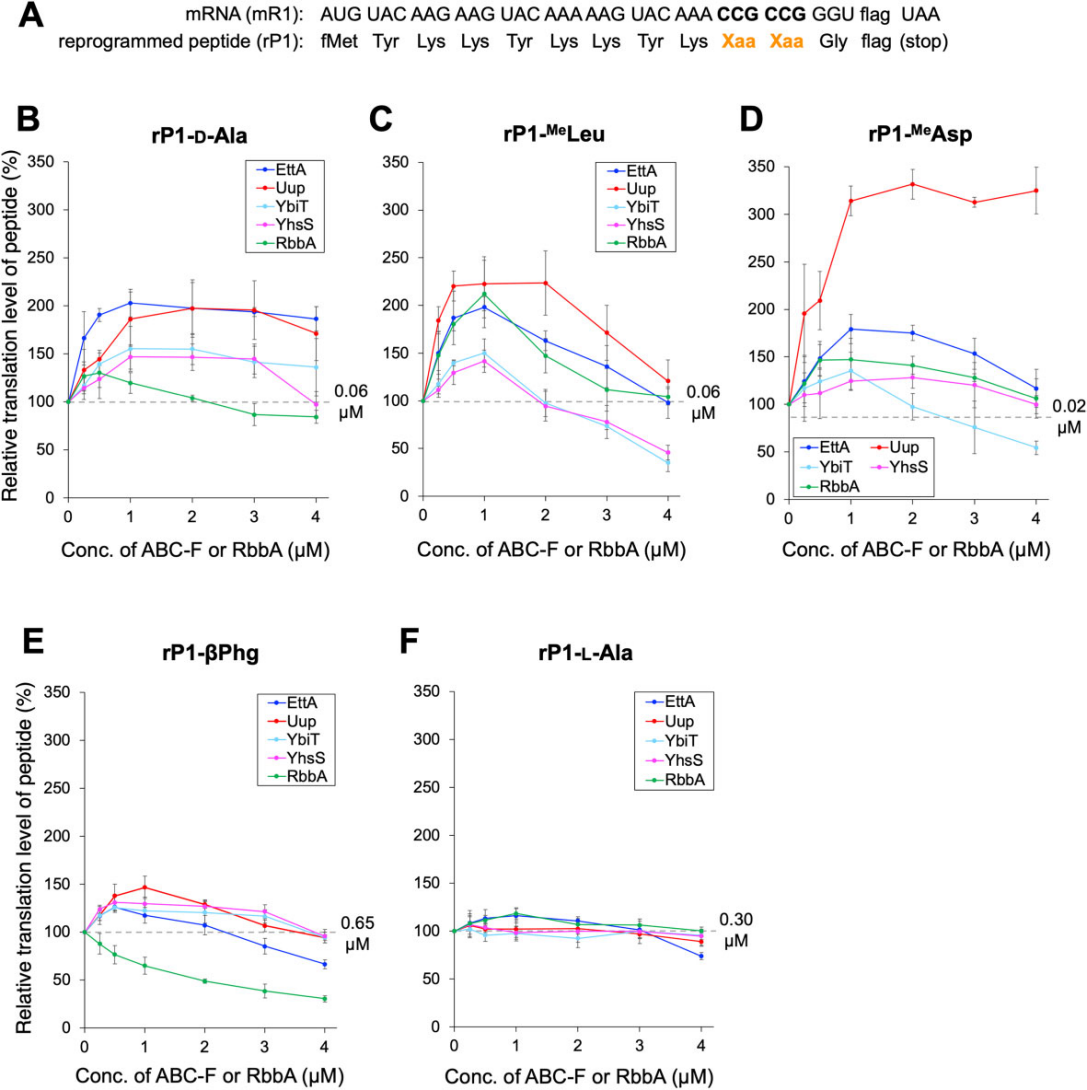
Ribosomal translation of model peptides containing consecutive npAAs in the presence of ABC-F proteins and RbbA. (**A**) Sequences of mRNA (mR1) and the corresponding peptide (rP1) used in this experiment. Xaa stands for arbitrary npAAs introduced at the CCG codons using precharged npAA-tRNAs. The amino acid sequence of “flag” is Asp–Tyr–Lys–Asp–Asp–Asp–Asp–Lys. (B–F) Quantification of relative translation levels of rP1 peptides containing npAAs. d-Ala (**B**), ^Me^Leu (**C**), ^Me^Asp (**D**), βPhg (**E**), and l-Ala (**F**) were introduced at the two consecutive CCG codons of mR1. Concentrations of EttA, Uup, YbiT, YhsS, and RbbA in the translation system were varied in a range of 0–4 μM. Error bars, standard deviation (SD), determined by three independent experiments. See also Supplementary Fig. S4A–E for raw tricine SDS–PAGE results. The absolute translation levels of the peptides in the absence of ABC-F and RbbA are shown at the right side of the dotted lines, which correspond to 100% relative translation level. For instance, in the case of rP1-d-Ala, the 100% relative translation level corresponds to 0.06 μM.

To evaluate the effects of ABC-F proteins and RbbA on the ribosomal incorporation of npAAs, we tested three types of npAAs as follows: (i) d-α-amino acid, e.g. d-Ala, (ii) *N*-methyl-l-α-amino acid, e.g. ^Me^Leu and ^Me^Asp, and (iii) β-amino acid, e.g. βPhg (Fig. 1). These npAAs were precharged on tRNA^Pro1E2^_CGG_ (Supplementary Fig. S2A, tRNA^Pro1E2^ bearing CGG anticodon) using dFx, a flexizyme variant, and introduced into a model peptide rP1 at two consecutive CCG codons of a template mRNA, mR1, using the FIT system (Fig. 2A) [61]. The FIT system was customized to contain five pAAs (Met, Tyr, Lys, Asp, and Gly) and the corresponding aminoacyl-tRNA synthetases. The other pAAs and their aminoacyl-tRNA synthetases were excluded. We used 3 μM of IF2, 0.1 μM of EF-G, 20 μM of EF-Tu, and 5 μM of EF-P for the npAA incorporation. These concentrations had been optimized for d-α- and β-amino acid incorporation in another study [4, 36, 62]. To incorporate ^Me^Asp, 5 μM of EF-Sep—an engineered EF-Tu variant—was added to promote the accommodation of negatively charged ^Me^Asp-tRNA onto the ribosomal A-site [63]. Concentrations of EttA, Uup, YbiT, YhsS, and RbbA were varied in a range of 0–4 μM (Fig. 2B–E). The identities of the translated peptides were confirmed by MALDI-TOF MS (Supplementary Fig. S3). For peptide quantification, translation was performed in the presence of [^14^C]-labeled Asp in place of cold Asp and the resulting peptides were separated by tricine SDS–PAGE and quantified by autoradiography (Supplementary Fig. S4). The absolute translation levels of rP1-d-Ala, rP1-^Me^Leu, rP1-^Me^Asp, and rP1-βPhg in the absence of ABC-F and RbbA were determined to be 0.06, 0.06, 0.02, and 0.65 μM, respectively (Fig. 2B–E). These levels were defined as 100% relative translation level for each peptide (Fig. 2B–E, indicated by dotted lines).

During d-Ala incorporation, 0.5–2 μM EttA, Uup, YbiT, YhsS, and RbbA significantly enhanced the translation level of the resulting peptide, rP1-d-Ala, compared with that in the absence of these proteins (Fig. 2B and Supplementary Figs S3A and S4A, translation levels were enhanced by 2.0-fold, 2.0-fold, 1.6-fold, 1.5-fold, and 1.3-fold with 1 μM EttA, 2 μM Uup, 1 μM YbiT, 1 μM YhsS, and 0.5 μM RbbA, respectively). However, a further increase in their concentrations at >2 μM gradually decreased the translation level of rP1-d-Ala. In ^Me^Leu and ^Me^Asp incorporation, this tendency was more obvious; the translation levels of rP1-^Me^Leu and rP1-^Me^Asp peaked with 1–2 μM of ABC-F or RbbA and significantly decreased at higher concentrations, indicating that high concentrations of these proteins were harmful for translation (Fig. 2C and Supplementary Figs S3B and S4B, translation levels were enhanced by 2.0-fold, 2.2-fold, 1.5-fold, 1.4-fold, and 2.1-fold for rP1-^Me^Leu with 1 μM EttA, 2 μM Uup, 1 μM YbiT, 1 μM YhsS, and 1 μM RbbA, respectively; Fig. 2D and Supplementary Figs S3B and S4C, 1.8-fold, 3.3-fold, 1.4-fold, 1.3-fold, and 1.5-fold for rP1-^Me^Asp with 1 μM EttA, 2 μM Uup, 1 μM YbiT, 2 μM YhsS, and 1 μM RbbA, respectively). The exception was ^Me^Asp incorporation with high Uup concentrations, where the rP1-^Me^Asp level did not decrease. Uup was more effective than the other proteins at promoting ^Me^Asp and ^Me^Leu incorporation, showing the advantage of using Uup in introduction of these amino acids. In βPhg incorporation, EttA, Uup, YbiT, and YhsS significantly enhanced the translation level of the resulting peptide, rP1-βPhg, in the presence of 0.5 or 1 μM proteins compared with that in their absence (Fig. 2E and Supplementary Figs S3C and S4D, 1.3-fold, 1.5-fold, 1.3-fold, and 1.3-fold with 0.5 μM EttA, 1 μM Uup, 0.5 μM YbiT, and 0.5 μM YhsS, respectively). By contrast, RbbA showed a significant decrease in translation levels even at low concentrations, such as 0.25, 0.5, and 1 μM (Fig. 2E and Supplementary Fig. S4D, 0.9-fold, 0.8-fold, and 0.7-fold with 0.25, 0.5, and 1 μM RbbA, respectively) and drastically reduced at higher concentrations (0.5–0.3-fold with 2–4 μM). Why translation is inhibited at high protein concentrations is unclear; however, we observed a similar tendency while titrating the concentration of EF-P for d-α- and β-amino acid incorporation [36, 62]. This is likely because EF-P remains occupying the ribosomal E-site even after peptidyl transfer is complete when concentration is high (>5 μM), thereby inhibiting the translocation of deacyl-tRNA from P-site to E-site. Because ABC-F and RbbA are E-site binders, similar tendencies could be observed at high concentrations for the same reason. Biochemical and structural analyses are warranted to verify these findings.

As the model peptide rP1 contains several Lys and Asp residues, nascent peptide-dependent translation arrest could be caused by not only npAAs but also these Lys and Asp residues. However, introduction of positively charged amino acids, such as Lys, was necessary for the efficient ionization of peptides in MALDI-TOF MS. Asp was introduced at the C-terminal Flag-tag sequence (Asp–Tyr–Lys–Asp–Asp–Asp–Asp–Lys) for Flag purification of peptides after translation and for [^14^C] labeling of peptides. To verify this possibility, we performed a control experiment, where two consecutive l-Ala residues were introduced at CCG codons instead of npAA using tRNA^Pro1E2^_CGG_ to synthesize rP1-l-Ala (Fig. 2F and Supplementary Figs S3E and S4E). EttA and RbbA slightly enhanced the translation level of rP1-l-Ala, but Uup, YbiT, and Yhs did not (Fig. 2F, 1.2-fold with 1 μM EttA or RbbA). Given that EttA alleviates translation arrest triggered by polyacidic sequences [53, 54], this result indicates that the Asp cluster in the Flag-tag sequence of rP1 caused modest translation arrest, which was resolved by adding EttA to the translation system. Because EttA addition resulted in a higher enhancement of rP1 translation levels for the introduction of d-Ala, ^Me^Leu, ^Me^Asp, and β-Phg (2.0-fold, 2.0-fold, 1.8-fold, and 1.3-fold, respectively) than that of l-Ala (1.2-fold), it is likely that the introduction of the npAAs caused more severe translation arrest than that of the Asp residues and its alleviation by EttA was effective for enhancing the translation levels of the resulting peptides. The addition of RbbA resulted in a higher enhancement of rP1 levels for d-Ala, ^Me^Leu, and ^Me^Asp incorporation (1.3-fold, 2.1-fold, and 1.5-fold, respectively). Because YbiT, a protein effective for polybasic sequences, did not enhance the translation level of rP1-l-Ala, it is likely the Lys cluster of this peptide did not cause translation arrest.

**Figure 3.**
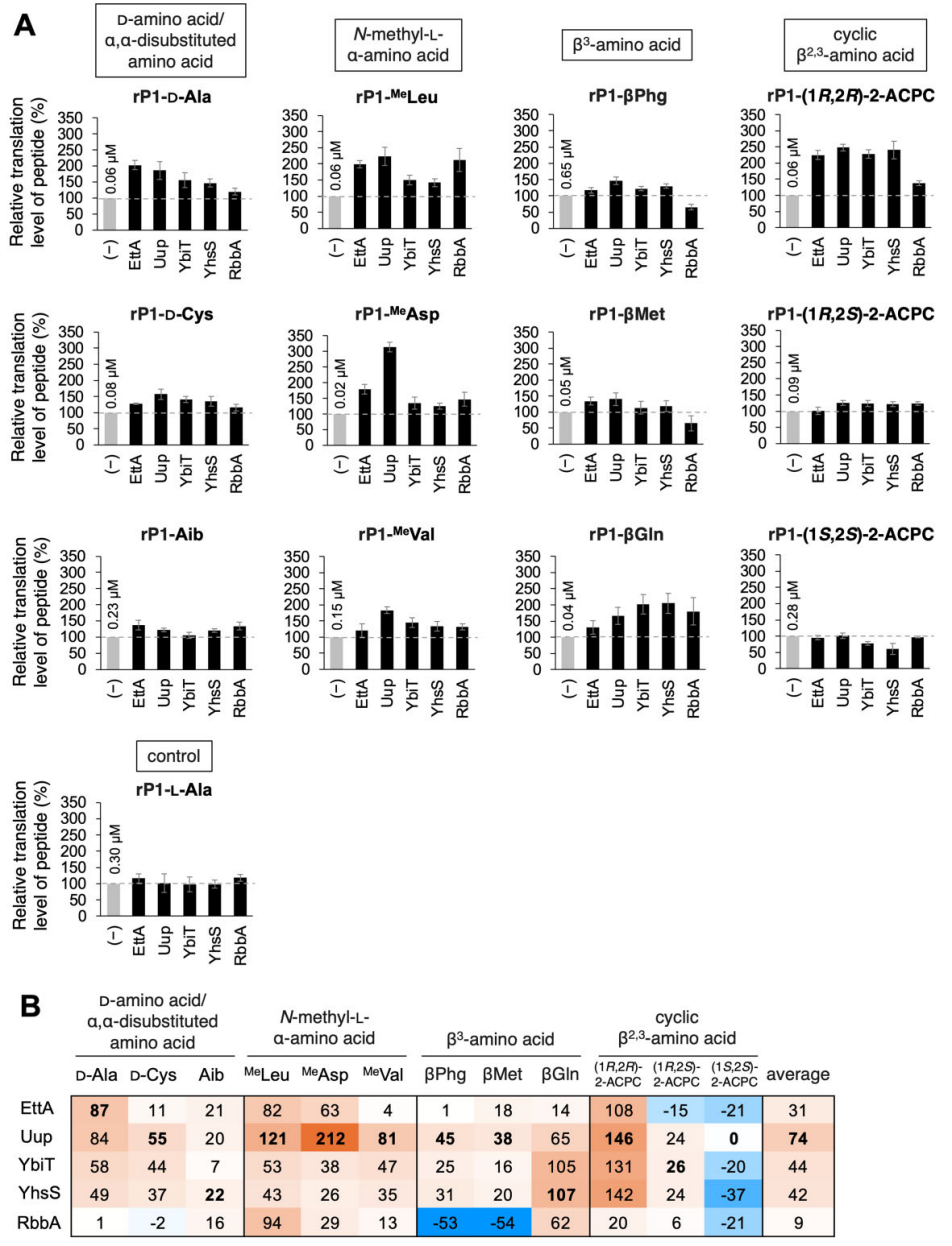
Ribosomal incorporation of various npAAs into rP1 in the presence of ABC-F proteins and RbbA. (**A**) Introduction of 12 types of npAAs and l-Ala into rP1 in the presence of 1 μM of EttA, Uup, YbiT, YhsS, or RbbA and quantification of relative translation levels. (−) indicates that ABC-F and RbbA were not added. The absolute translation levels of the peptides are shown for (−). Note that the results for d-Ala, ^Me^Leu, ^Me^Asp, βPhg, and l-Ala are excerpted from Fig. 2B–F. Error bars, SD, determined by three independent experiments. See also Supplementary Fig. S4A–I for raw tricine SDS–PAGE results. (**B**) Estimation of “% enhancement” values calculated by subtracting the relative translation level of rP1-l-Ala from that of rP1-npAA in the presence of 1 μM ABC-F or RbbA. The highest values for the respective substrates are indicated in bold.

To further validate the role of ABC-F and RbbA in the translation of various npAAs, we compared the translation level of rP1 in introducing 12 types of npAAs in the presence of EttA, Uup, YbiT, YhsS, or RbbA (Fig. 3A and Supplementary Figs S3 and S4F–I). In this experiment, the concentrations of these protein factors were fixed at 1 μM because the translation levels of rP1 with d-Ala, ^Me^Leu, ^Me^Asp, and βPhg peaked in the presence of 1 μM proteins in most of the combinations tested in Fig. 2B–F. As representatives of d-α-amino acid and α,α-disubstituted amino acid, we tested d-Cys and Aib in addition to d-Ala. These three substrates were significantly enhanced by all proteins, except for the combination of Aib and YbiT, whose enhancement was less significant (Figs 1A and 3A, 1.1-fold for rP1-Aib + YbiT and 1.2–2.0-fold for the other combinations). For *N*-methyl-l-α-amino acids, incorporation of ^Me^Leu, ^Me^Asp, and ^Me^Val was significantly enhanced by all proteins (Figs 1B and 3A, 1.2–3.1-fold). No significant inhibitory effects were observed by any of the five proteins for d-amino and *N*-methyl-l-α-amino acid incorporation unlike that observed for β-amino acid incorporation.

In β-amino acid incorporation, βMet, βGln, (1*R*,2*R*)-2-aminocyclopentane carboxylic acid [(1*R*,2*R*)-2-ACPC], (1*R*,2*S*)-2-ACPC, and (1*S*,2*S*)-2-ACPC were tested as representatives of β-amino acids in addition to βPhg (Fig. 1C and D). We have previously demonstrated that EF-P enhanced the incorporation of β^3^-amino acids, such as βPhg, βMet, and βGln, whereas the incorporation of cyclic β^2,3^-amino acids, such as 2-ACPC, was differently regulated by EF-P depending on stereochemical configurations; (1*R*,2*S*)-2-ACPC was enhanced and (1*R*,2*R*)-2-ACPC and (1*S*,2*S*)-2-ACPC were inhibited by EF-P. Therefore, we evaluated side chain or stereochemistry dependencies of translation regulation mediated by EttA, Uup, YbiT, YhsS, and RbbA by testing these substrates. Regarding β^3^-amino acids, the incorporation of βPhg and βMet was enhanced by EttA, Uup, YbiT, and YhsS (Fig. 3A, 1.2–1.5-fold for βPhg; 1.1–1.4-fold for βMet) and inhibited by RbbA (Fig. 3A, 0.7- and 0.6-fold). By contrast, the incorporation of βGln was enhanced by all the proteins, including RbbA (Fig. 3A, 1.3–2.1-fold). As for cyclic β^2,3^-amino acids, the incorporation of (1*R*,2*R*)-2-ACPC was significantly enhanced by all the proteins (Fig. 3A, 1.4–2.5-fold), whereas that of (1*S*,2*S*)-2-ACPC was not enhanced or negatively regulated (Fig. 3A, 0.8- and 0.6-fold with YbiT and YhsS). The regulation of (1*R*,2*S*)-2-ACPC incorporation was modest with any of these proteins (Fig. 3A, 1.0–1.3-fold).

EttA and RbbA are likely involved in the alleviation of translation arrest caused by npAAs and the Asp cluster of rP1. Therefore, to evaluate the translation enhancement effects solely attributed to the alleviation of npAA-derived translation arrest, we estimated “% enhancement” value, which was calculated by subtracting the relative translation level of rP1-l-Ala from that of rP1-npAA in the presence of 1 μM ABC-F or RbbA (Fig. 3B). In the average of the 12 substrates, Uup exhibited the highest % enhancement value of 74% (Fig. 3B, 31%, 44%, 42%, and 9% for EttA, YbiT, YhsS, and RbbA, respectively). As Uup is reportedly involved in alleviating the translation arrest caused by Pro-rich sequences [53], it is possible that Uup promotes the translation of nascent peptides containing multiple *N*-methyl-l-α-amino acids. Consistently, Uup was particularly efficient in enhancing the translation levels of rP1-^Me^Leu, ^Me^Asp, and ^Me^Val (Fig. 3B, 121%, 212%, and 81% enhancement, respectively, with 1 μM Uup).

### Combinations of EttA, Uup, and RbbA in the translation of rP1-^Me^Leu

**Figure 4.**
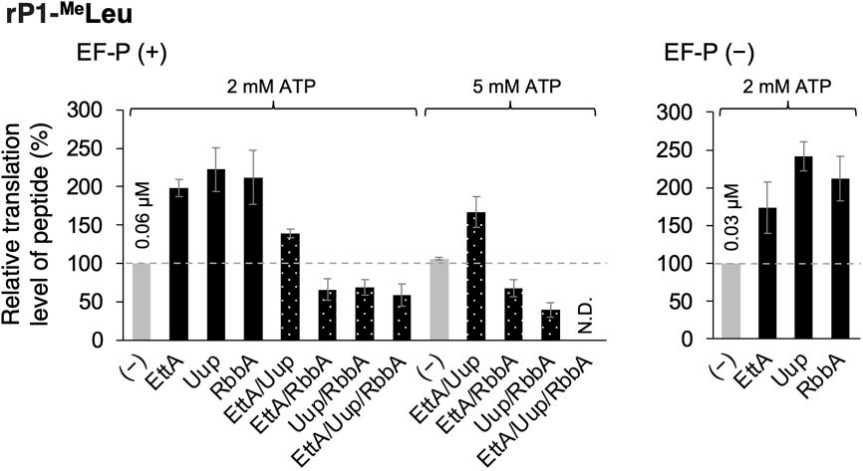
Combinations of EttA, Uup, and RbbA in the presence and absence of EF-P. Ribosomal incorporation of ^Me^Leu into rP1 demonstrated with combinations of EttA, Uup, and RbbA and their translation levels were quantified. (−) indicates that EttA, Uup, and RbbA were not added. 2 mM or 5 mM ATP was tested in this analysis; the standard ATP concentration in the FIT system is 2 mM as described in the “Materials and methods” section. The absolute translation levels of the peptides are shown for (−) with 2 mM ATP. For EF-P (+) translation, 5 μM EF-P was added.

These results prompted us to test combinations of ABC-F and RbbA to further enhance ribosomal incorporation of multiple npAAs. We focused on EttA, Uup, and RbbA because Uup showed the highest enhancement effects, whereas EttA and RbbA showed unique tendencies depending on substrate type. The translation level of rP1-^Me^Leu was evaluated with combinations of EttA/Uup, EttA/RbbA, Uup/RbbA, and EttA/Uup/RbbA, where the concentration of each protein was fixed to 1 μM, and compared with their absence or their individual use (Fig. 4 and Supplementary Fig. S4J, K, (−), EttA, Uup, RbbA, EttA/Uup, EttA/RbbA, Uup/RbbA, and EttA/Uup/RbbA). First, translation was conducted in the presence of 5 μM EF-P and 2 mM ATP. The EttA/Uup combination resulted in lower translation level of rP1-^Me^Leu compared with when EttA or Uup was used alone, indicating that their combinatorial use did not further enhance the translation levels of peptides in an additive manner (Fig. 4, left, and Supplementary Fig. S4J). Consistently, combinations of EttA/RbbA, Uup/RbbA, and EttA/Uup/RbbA resulted in lower or comparable translation levels of peptides than when the proteins were used individually. This is likely because these proteins bind to the same site of the ribosome to alleviate translation arrest caused by the same nascent peptide context associating with the ribosomal tunnel, and therefore, compete with each other to bind to the same site when added to the translation system at once. Since ABC-F and RbbA are ATPses, we next tested the effect of increasing ATP concentration to 5 mM. The combinations of EttA/Uup, EttA/RbbA, and Uup/RbbA resulted in comparable rP1-^Me^Leu levels to 2 mM ATP, whereas the combination of EttA/Uup/RbbA completely inhibited the translation of rP1-^Me^Leu to an undetectable level (Fig. 4, left, and Supplementary Fig. S4J). This is likely because, in the presence of 5 mM ATP, higher populations of these proteins became ATP-bound active forms and more frequently competed with each other at the E site.

**Figure 5.**
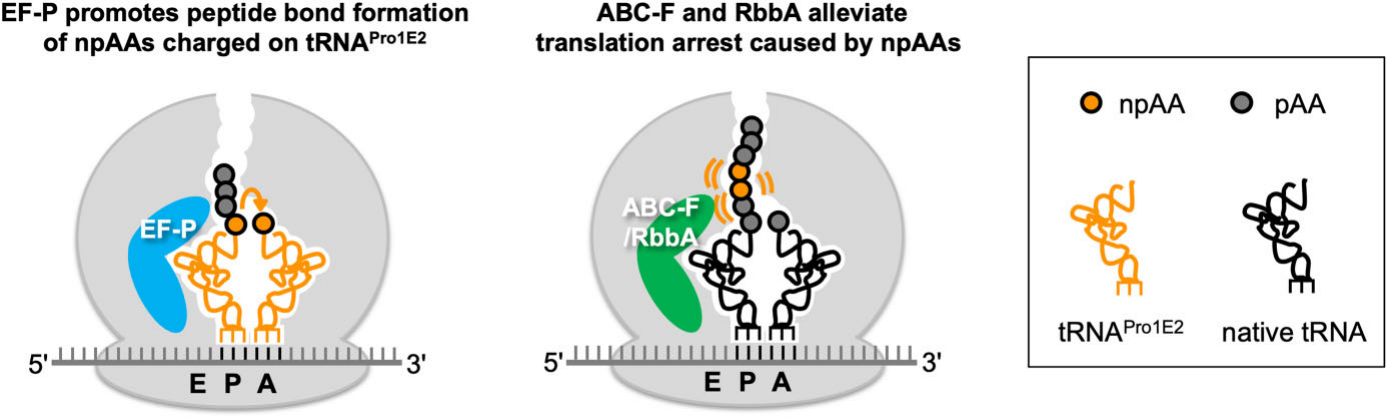
Illustration of the roles of the E-site binder proteins, EF-P, ABC-F, and RbbA. EF-P recognizes the specific D-arm motif of the P-site tRNA^Pro1E2^ and binds to a site between E-site and P-site, thereby promoting peptidyl transfer reaction between the P-site peptidyl-tRNA and A-site aminoacyl-tRNA (left). ABC-F proteins and RbbA bind to the ribosomal E-site upon translation arrest caused by nascent peptide contexts to resolve ribosomal stalling (right). Because EF-P and ABC-F/RbbA bind to different states of the ribosome, they do not compete at the ribosomal E-site.

We next conducted the translation of rP1-^Me^Leu in the absence of EF-P with 1 μM EttA, Uup, or RbbA. There was no significant difference between EF-P (+) and EF-P (−) in the enhancement of relative translation levels by EttA, Uup, and RbbA, indicating that these factors do not compete with EF-P, although EF-P is also an E-site binder (Fig. 4, right). This is likely because EF-P binds to the ribosome only when tRNA^Pro1E2^ is at the P-site. tRNA^Pro1E2^ is designed to have the specific D-arm motif that is recognized by EF-P to promote peptidyl transfer between the npAA charged on tRNA^Pro1E2^ at the P-site and the next amino acid charged on the A-site aminoacyl-tRNA (Fig. 5, left). By contrast, ABC-F and RbbA would bind to the ribosome when translation arrest is caused by a nascent peptide containing multiple npAAs (Fig. 5, right). Under these circumstances, because npAAs have been incorporated into the nascent peptide chain and tRNA^Pro1E2^ has exited, EF-P does not bind the ribosome, and therefore, EF-P would not compete with ABC-F and RbbA. Comparing the absolute translation levels of rP1-^Me^Leu in the absence and presence of EF-P, there is approximately two-fold enhancement by EF-P (Fig. 4, 0.03 and 0.06 μM in the absence and presence of EF-P, respectively, without ABC-F and RbbA). Moreover, adding 1 μM EttA, Uup, or RbbA also resulted in twofold enhancement, where the absolute rP1-^Me^Leu level is ∼0.12 μM in the presence of EF-P. Therefore, the combination of the tRNA^ProE2^/EF-P system with EttA, Uup or RbbA enables approximately four-fold enhancement of the rP1-^Me^Leu translation level in total, showing the usefulness of this combination in introducing npAAs.

### Ribosomal incorporation of multiple types of npAAs in the presence of Uup

**Figure 6.**
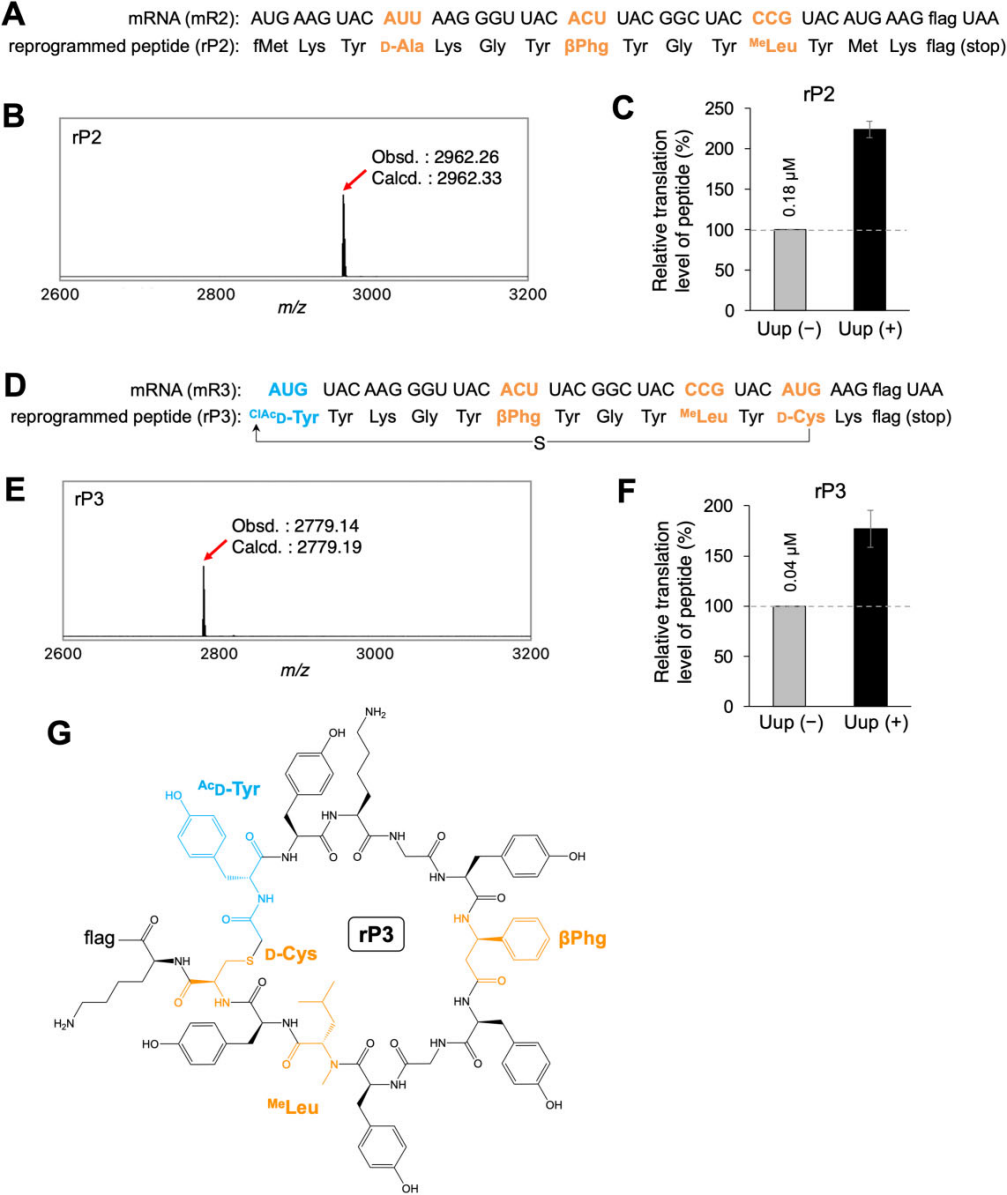
Ribosomal incorporation of multiple npAAs into model peptides in the presence of Uup. (**A**and**D**) Sequences of mRNAs (mR2 and mR3) and the corresponding peptides (rP2 and rP3) used. The amino acid sequence of “flag” is Asp–Tyr–Lys–Asp–Asp–Asp–Asp–Lys. Identification of the translated peptides, rP2 (**B**) and rP3 (**E**), by MALDI-TOF MS. Translation was performed in the presence of 1 μM Uup. Arrows indicate the monovalent ions ([M + H]^+^) of the desired products. “Obsd.” and “Calcd.” indicate observed and calculated *m*/*z* values, respectively. Quantification of relative translation levels of the peptides rP2 (**C**) and rP3 (**F**). Translation was performed in the presence or absence of 1 μM Uup. Error bars, SD, determined by three independent experiments. The absolute translation levels of the peptides are shown for (−). See also Supplementary Fig. S4L for raw tricine SDS–PAGE results. (**G**) Chemical structure of the rP3 peptide. The sulfhydryl group of d-Cys spontaneously reacts with the N-terminal ClAc group to form a thioether bond, yielding a macrocyclic structure.

This study aimed to promote the efficient translation of difficult peptide sequences containing multiple types of npAAs, such as d-α-, *N*-methyl-l-α-, and β-amino acids, by taking advantage of ABC-F proteins. We chose rP2 and rP3 as model peptide sequences (Fig. 6A and D). rP2 is a linear peptide containing d-Ala, βPhg, and ^Me^Leu. These amino acids were precharged on tRNA^Pro1E2^_GAU_, tRNA^Pro1E2^_GGU_, and tRNA^Pro1E2^_CGG_ by using dFx and introduced at AUU, ACU, and CCG codons of mR2, respectively. Because Uup showed the highest % enhancement in average for incorporation of the 12 substrates tested in this study (Fig. 3B, 74% in average), we decided to add 1 μM Uup to the translation system. The identity of the translated rP2 peptide was confirmed by MALDI-TOF MS (Fig. 6B). The translation level of rP2 was enhanced by 2.2-fold in the presence of 1 μM Uup compared with the absence (Fig. 6C and Supplementary Fig. S4L).

In the translation of rP3, ^ClAc^ d-Tyr, βPhg, ^Me^Leu, and d-Cys were charged on tRNA^iniP^_CAU_, tRNA^Pro1E2^_GGU_, tRNA^Pro1E2^_CGG_, and tRNA^Pro1E2^_CAU_ and introduced at the initiator AUG, elongator ACU, CCG, and AUG codons of mR3, respectively (Fig. 6D). tRNA^iniP^_CAU_ is an engineered initiator tRNA that has the specific D-arm motif recognized by EF-P to enhance npAA incorporation in the initiation event (Supplementary Fig. S2B) [64]. Upon translation, the N-terminal chloroacetyl group of ^ClAc^ d-Tyr spontaneously reacts with the thiol group of the downstream d-Cys to form a thioether bond, resulting in the macrocyclization of peptide (Fig. 6G). The identity of the translated rP3 was confirmed by MALDI-TOF MS (Fig. 6E). The translation level of rP3 was enhanced by 1.8-fold in the presence of 1 μM Uup compared with the absence (Fig. 6F and Supplementary Fig. S4L). These results indicate the difficulty of translating peptides containing multiple npAAs, such as rP2 and rP3, likely due to translation arrest, which can be overcome by utilizing Uup.

## Discussion

Here, we have demonstrated that ABC-F proteins and RbbA can enhance translation level of peptides containing multiple or consecutive npAAs. These results indicate the possibility that introduction of npAAs causes nascent peptide-dependent translation arrest and/or ribosome destabilization, which could be circumvented by adding ABC-F proteins and RbbA to the translation system. Although how ABC-F proteins resolve translation arrest and ribosome destabilization caused by npAAs has not been clarified yet, the mechanism how these proteins rescue ribosomes stalled by antibiotics has been suggested [47–50, 52]. Upon binding of the ATP (or NTP)-bound ABC-F to the ribosomal E-site, the interdomain linker between the two ATP-binding domains is inserted into the PTase center, thereby rearranging the PTase center and the P-site peptidyl-tRNA, due to which antibiotic is released from the ribosome and/or hindered from binding. Then, ATP hydrolysis triggers dissociation of ABC-F from the ribosome, while, in the case of YheS, it has been suggested that ATP hydrolysis is required for its arrest-releasing activity [53]. Such rearrangements might be effective for resolving nascent peptide-dependent translation arrest and ribosome destabilization caused by npAAs as well as for the inhibition of antibiotic binding. On the other hand, RbbA also has two ATP-binding domains and binds to the ribosomal E-site [58], but its functional role is less clear. It has been suggested that RbbA induces structural rearrangement of the ribosome to promote the release of E-site deacyl-tRNA and accommodation of aminoacyl-tRNA onto A-site [58]. Therefore, it is possible that rearrangements of the ribosome and tRNA are critical for both RbbA- and ABC-F-mediated alleviation of translation arrest and ribosome destabilization. By contrast, these proteins differently regulated the translation levels of model peptides depending on npAA type; e.g. βPhg incorporation was upregulated by ABC-F but downregulated by RbbA, indicating that rearrangements of ribosome and tRNA occur differently by binding of these proteins to the E-site.

Among ABC-F and RbbA, Uup showed the highest translation promotion effect in average for the 12 npAA substrates tested in this study. Because Uup resolves translation arrest caused particularly by poly-Pro nascent peptides, it is possible that the mode of translation arrest caused by backbone-modifying npAAs is more similar to that caused by poly-Pro compared with those of other types, such as poly-Asp/Glu and poly-Arg/Lys. Results of the competition experiments revealed that ABC-F and RbbA compete for binding to the E-site and their combinatorial use resulted in a significantly lower translation enhancement effect (Fig. 4). Therefore, combinations of these proteins must not be used to enhance the translation levels of npAA-containing peptides. Given that Uup is most effective for the incorporation of npAAs, adding 1 μM Uup to the translation system is agreeable for applications that introduce multiple types of npAAs at once into diverse peptides, such as when constructing random peptide libraries. Because Uup did not compete with EF-P, although EF-P is an E-site binder, Uup is compatible with the engineered tRNA system using tRNA^Pro1E2^ in combination with EF-P to increase npAA incorporation. The introduction of several types of npAAs charged on tRNA^Pro1E2^ in the presence of Uup and EF-P resulted in clean and enhanced expression of model macrocyclic peptides containing four types of npAAs at once. These findings indicate the usefulness of this system in expressing macrocyclic peptide libraries bearing many npAAs and its application in mRNA display-based screening methods to identify bioactive peptides (Fig. 6).

## Supplementary Material

gkaf446_Supplemental_Files

## Data Availability

The data underlying this research are available in the article and in its online supplementary material.

## Supplementary data

Supplementary data is available at NAR online.
