# Supplementary material for: Promoting ribosomal incorporation of backbone-modifying nonproteinogenic amino acids into nascent peptides by ATP-binding cassette family-F proteins and EF-P
Source: Nucleic Acids Res. 2025 May 22;53(10):gkaf446. doi: 10.1093/nar/gkaf446 (PMC12096078; doi:10.1093/nar/gkaf446)
Supplement: gkaf446_Supplemental_Files [file gkaf446_supplemental_files.zip › 250418ABCF+caption_SI.pdf]

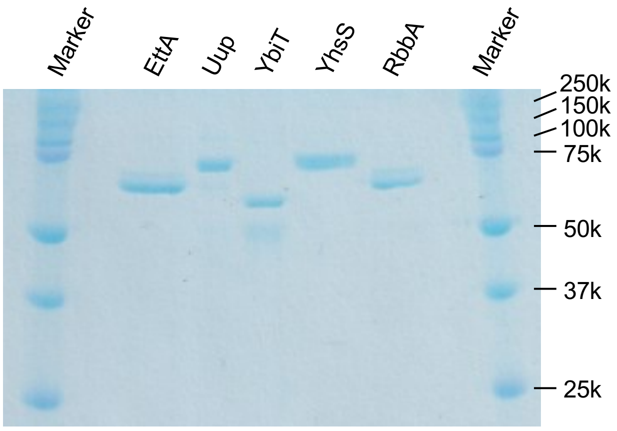

Supplementary Figure S1. SDS-PAGE analysis of the purified ABC-F proteins and RbbA. Molecular weights of EttA, Uup, YbiT, YhsS and RbbA are 62, 72, 60, 72 and 62 kDa, respectively. Precision Plus Protein Dual Color Standards (Bio-rad) were used as the marker. See also materials and methods section for the preparation of these proteins.

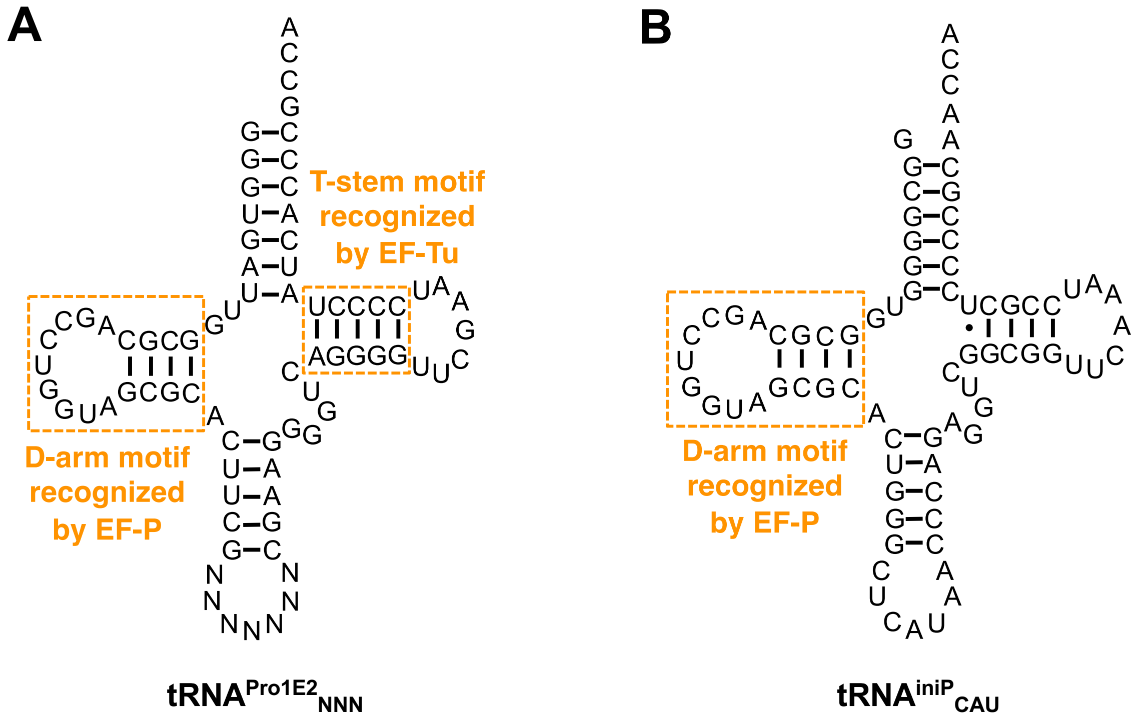

Supplementary Figure S2. Secondary structures of the engineered tRNAs used in this study. (A) $tRNA^{Pro1E2}$ used for npAA incorporation in elongation event. The anticodon loop sequence was changed accordingly to read cognate codons. See Supplementary Table S1 for the anticodon loop sequences. **(B)** $tRNA^{iniP}$ used for ^{ClAc}D-Tyr incorporation in initiation.

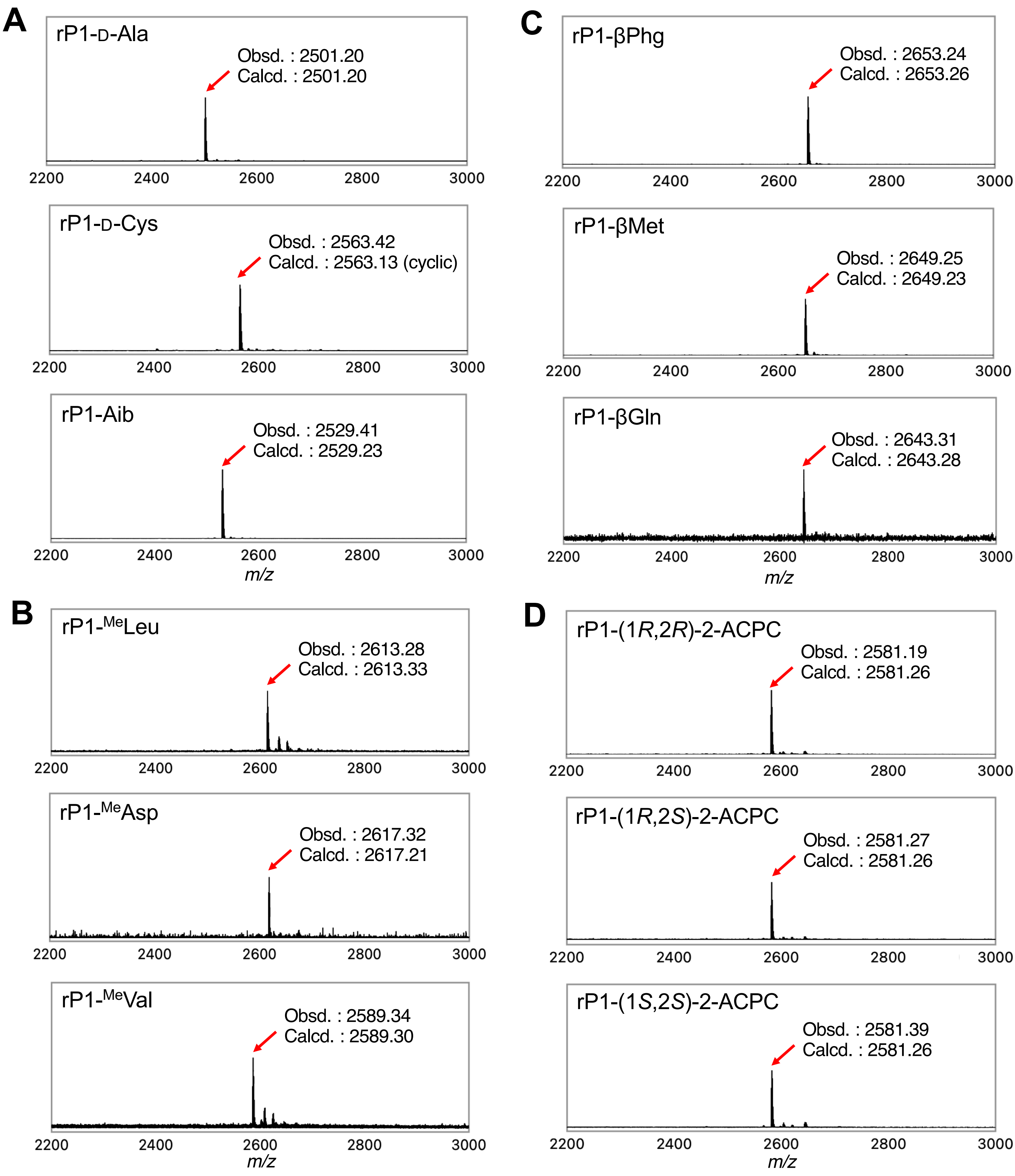

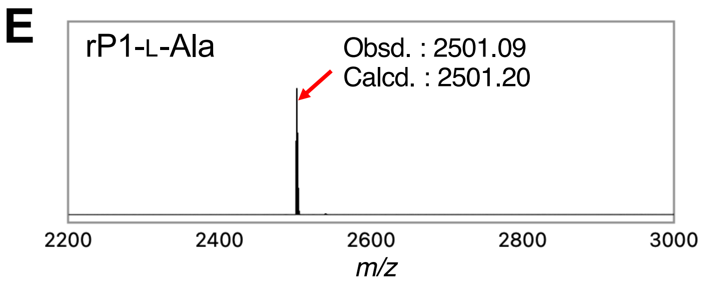

Supplementary Figure S3. MALDI-TOF MS of model peptides. (A−E) Introduction of D-amino acids and α,α-disubstituted amino acids (A), *N*-methyl-L-α-amino acids (B), β^3-amino acids (C), cyclic $\beta^{2,3}$-amino acids (D) and L-Ala (E) into peptide rP1 using $tRNA^{Pro1E2}_{CGG}$. 'Obsd.' and 'Calcd.' indicate observed and calculated $[M+H]^+$ values of the desired peptides, respectively.

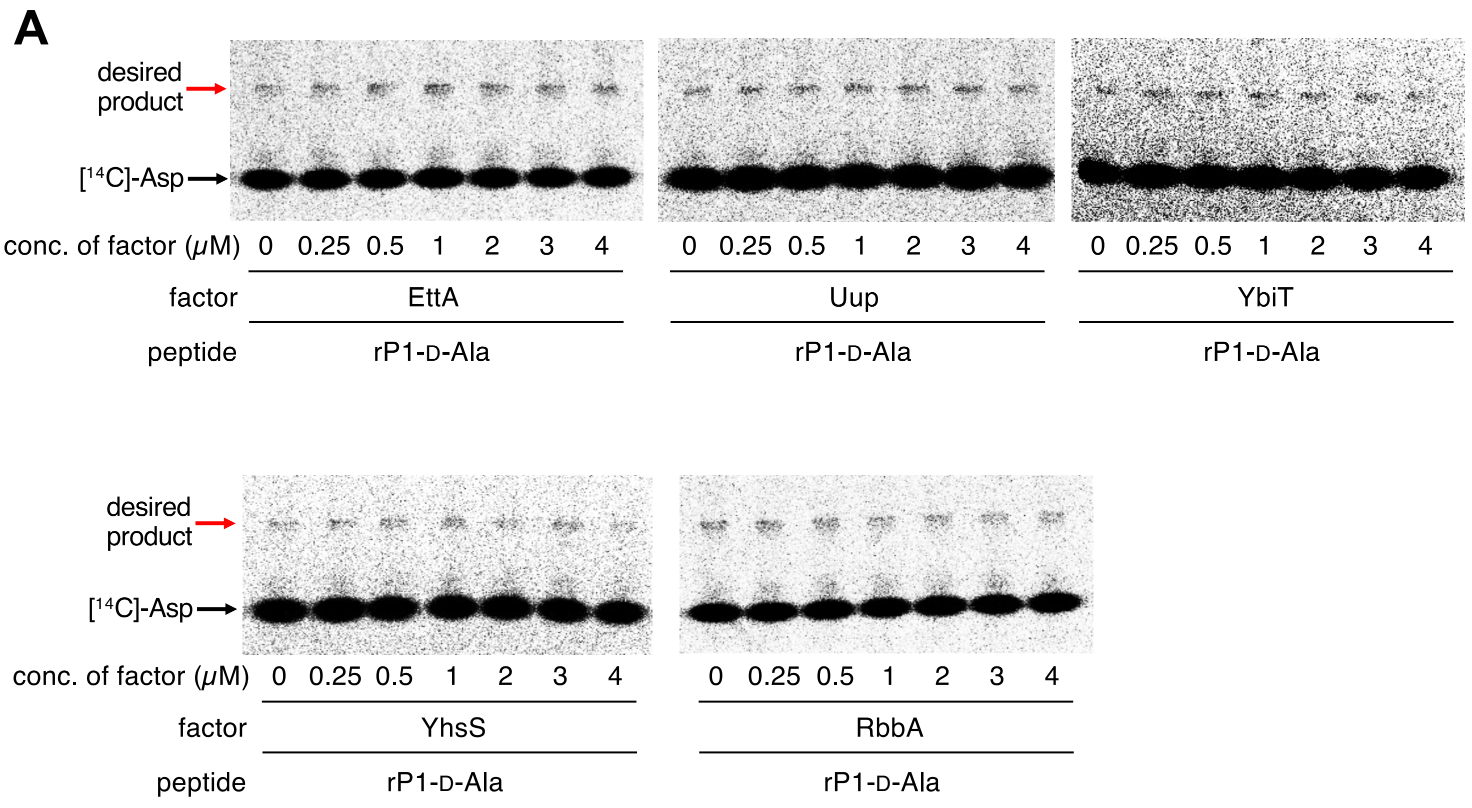

Supplementary Figure S4. Tricine SDS-PAGE analysis of translated peptides. (A–E) Incorporation of D-Ala (A), ^{Me}Leu (B), ^{Me}Asp (C), βPhg (D) and L-Ala (E) into peptide rP1 using tRNA$^{Pro1E2}_{CGG}$ in the presence of ABC-F proteins or RbbA. See also Figure 2B–F for the quantification of resulting peptides. **(F–I)** Incorporation of D-Cys and Aib (F), ^{Me}Val (G), βMet and βGln (H) and 2-ACPCs (I) into peptide rP1 using tRNA$^{Pro1E2}_{CGG}$ in the presence of 1 μM ABC-F proteins or RbbA. See also Figure 3A for the quantification. **(J)** Combinations of EttA, Uup and RbbA in ^{Me}Leu incorporation into rP1. The concentration of each protein factor was fixed to 1 μM. 5 μM EF-P was also added. 2 mM or 5 mM ATP was tested. See also Figure 4 left for the quantification. **(K)** ^{Me}Leu incorporation into rP1 in the absence of EF-P. 1 μM of EttA, Uup or RbbA was added to the translation system. See also Figure 4 right for the quantification. **(L)** Translation of model peptides rP2 and rP3 in the absence or presence of 1 μM Uup. 5 μM EF-P was also added. See also Figure 6C and 6F for the quantification. We performed three independent and replicated experiments for quantification; one representative experimental result is shown in this figure out of the three replicated experiments.

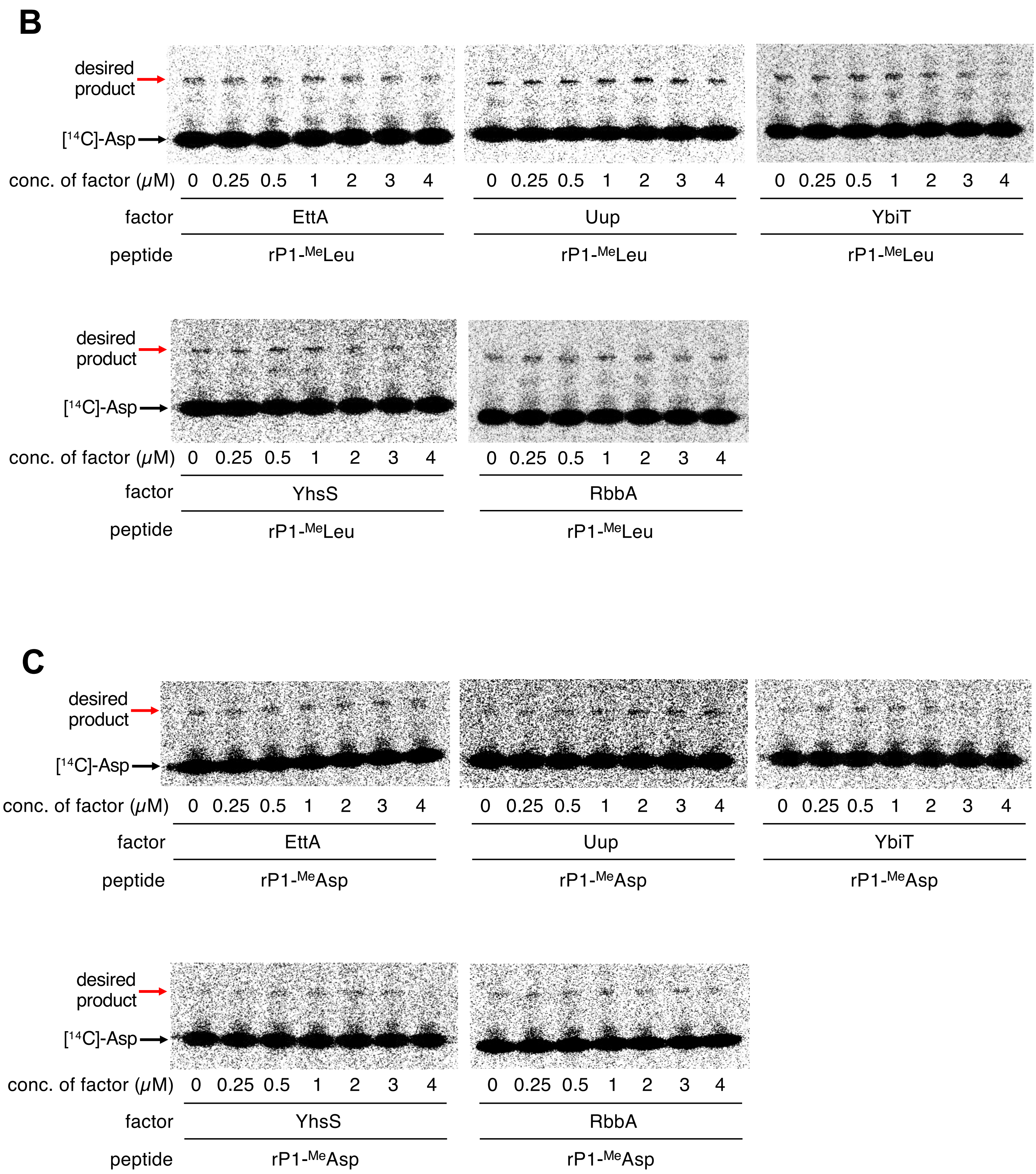

Supplementary Figure S4 continued.

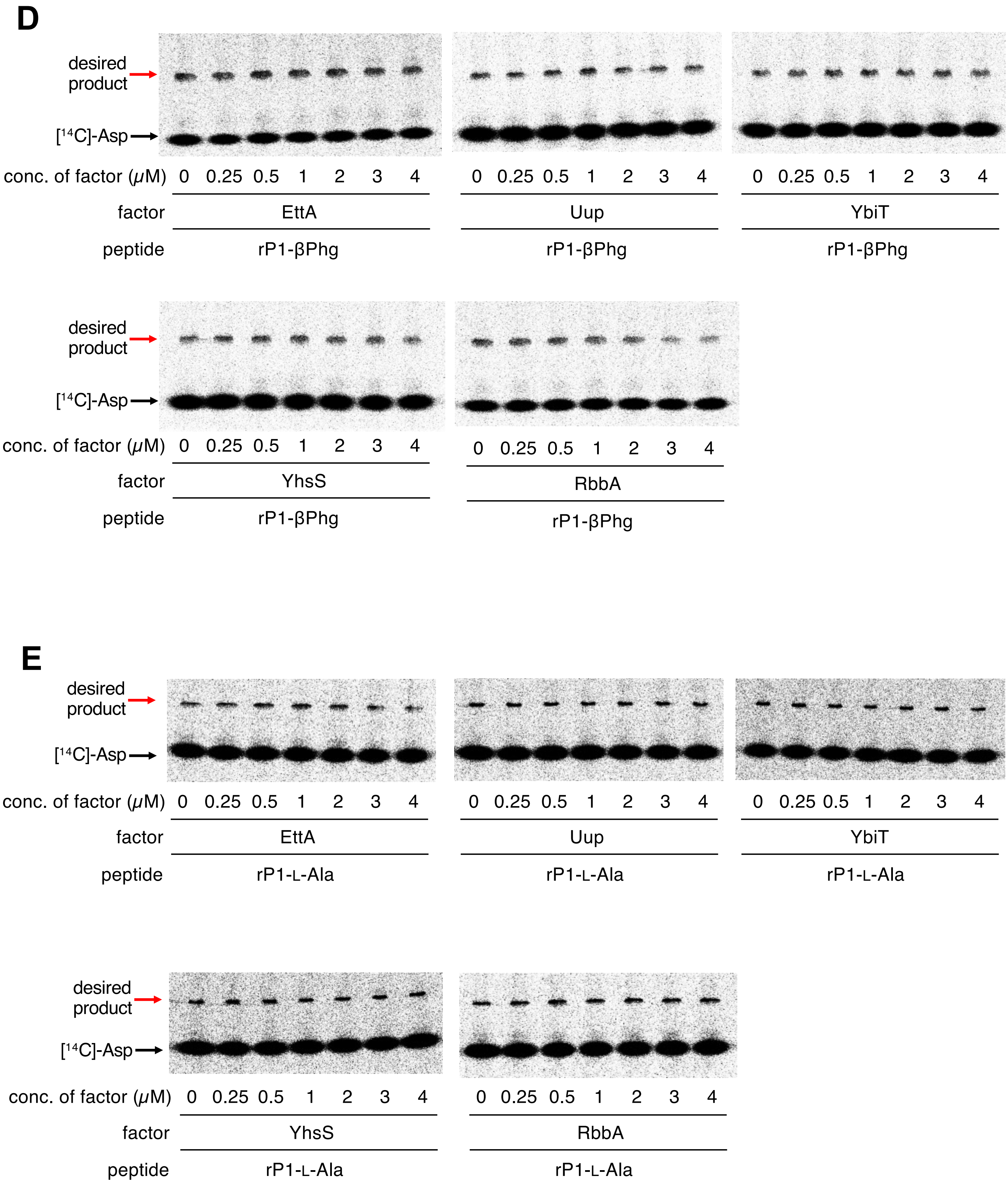

Supplementary Figure S4 continued.

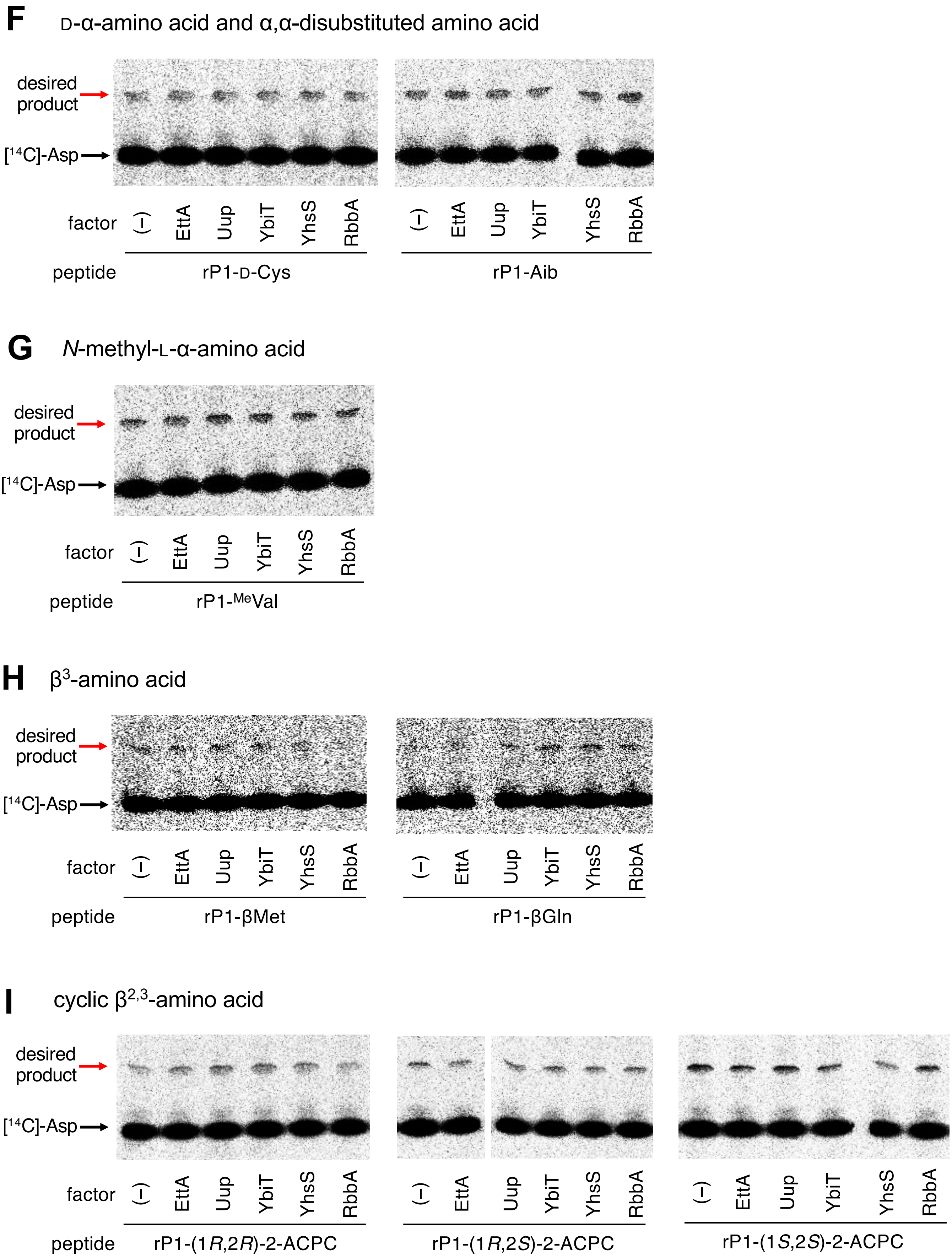

Supplementary Figure S4 continued.

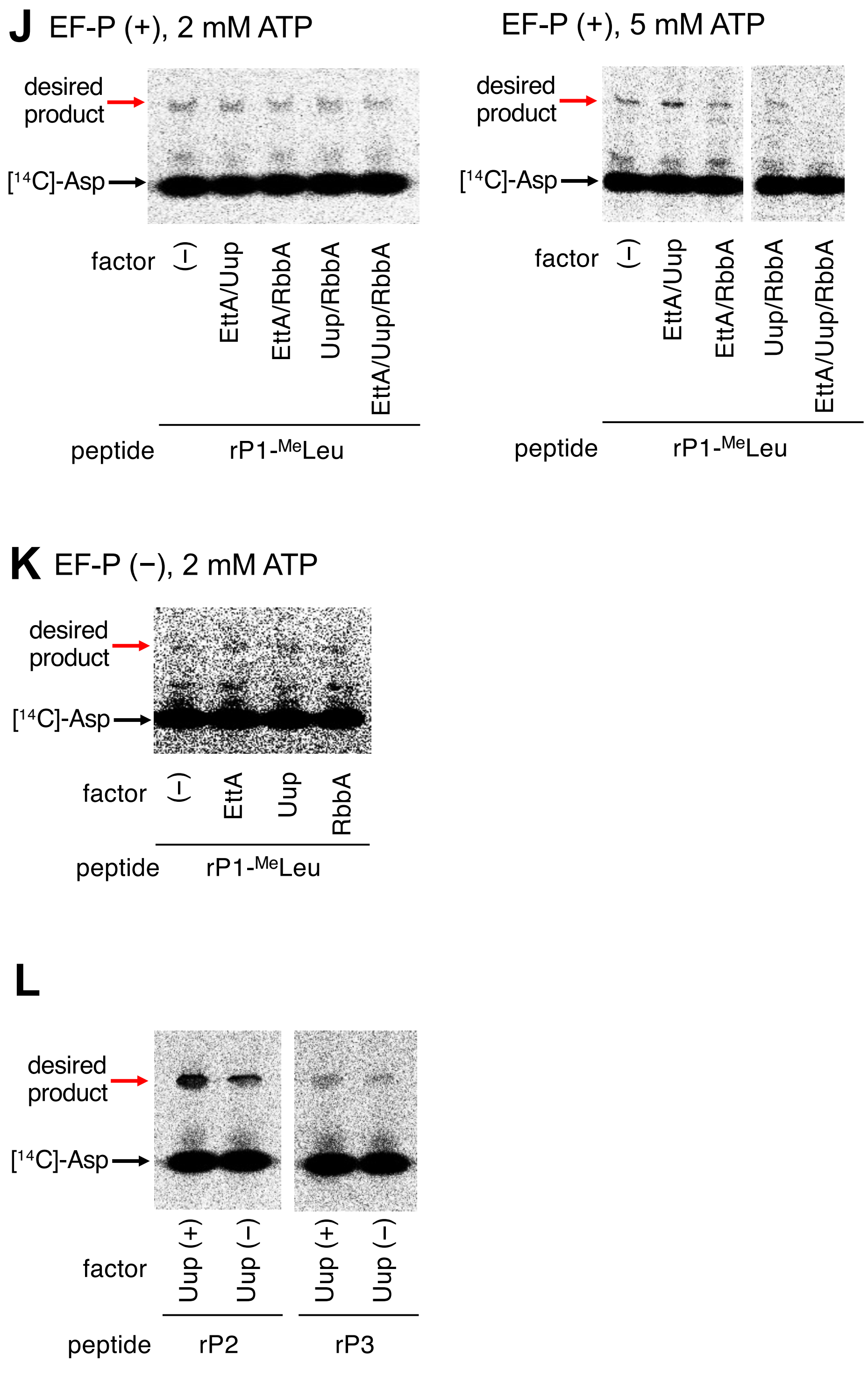

Supplementary Figure S4 continued.
